# Pediatric uveitis: Role of the pediatrician

**DOI:** 10.3389/fped.2022.874711

**Published:** 2022-08-01

**Authors:** Abhay Shivpuri, Inga Turtsevich, Ameenat Lola Solebo, Sandrine Compeyrot-Lacassagne

**Affiliations:** ^1^Rheumatology Department, Great Ormond Street Hospital for Children, London, United Kingdom; ^2^Biomedical Research Centre, Great Ormond Street Hospital for Children, London, United Kingdom; ^3^University College London (UCL) Great Ormond Street Institute of Child Health, London, United Kingdom

**Keywords:** uveitis, inflammation, pediatric uveitis, pediatrician, ocular inflammation

## Abstract

The challenges of childhood uveitis lie in the varied spectrum of its clinical presentation, the often asymptomatic nature of disease, and the evolving nature of the phenotype alongside normal physiological development. These issues can lead to delayed diagnosis which can cause significant morbidity and severe visual impairment. The most common ocular complications include cataracts, band keratopathy, glaucoma, and macular oedema, and the various associated systemic disorders can also result in extra-ophthalmic morbidity. Pediatricians have an important role to play. Their awareness of the various presentations and etiologies of uveitis in children afford the opportunity of prompt diagnosis before complications arise. Juvenile Idiopathic Arthritis (JIA) is one of the most common associated disorders seen in childhood uveitis, but there is a need to recognize other causes. In this review, different causes of uveitis are explored, including infections, autoimmune and autoinflammatory disease. As treatment is often informed by etiology, pediatricians can ensure early ophthalmological referral for children with inflammatory disease at risk of uveitis and can support management decisions for children with uveitis and possible underling multi-system inflammatory disease, thus reducing the risk of the development of irreversible sequelae.

## Introduction

Pediatric uveitis is rare condition, which accounts for 5–10% of all-age uveitis (1). Although variable within and across different countries, the estimated incidence of childhood uveitis in industrialized nations is ~4.3 per 100,000 with a prevalence of 27.9 per 100,000 (2).

Diagnosis of pediatric uveitis can be challenging due to insidious (chronic, persistent and recurrent) presentation, lack of symptom reporting in early childhood, and difficulties in examining young children. Pediatric uveitis is also characterized by frequent complications and comorbidities such as cataract, glaucoma or amblyopia (potentially irreversible failure to develop good vision).

The focus of this review is the role of the pediatrician in all stages, including recognition of the signs or symptoms (or lack thereof) of uveitis in the child at risk due to their rheumatological condition; advising on possible etiology; and co-managing systemic therapy in the child with uveitis and systemic disorders.

## Eye anatomy

**Figure 1:**
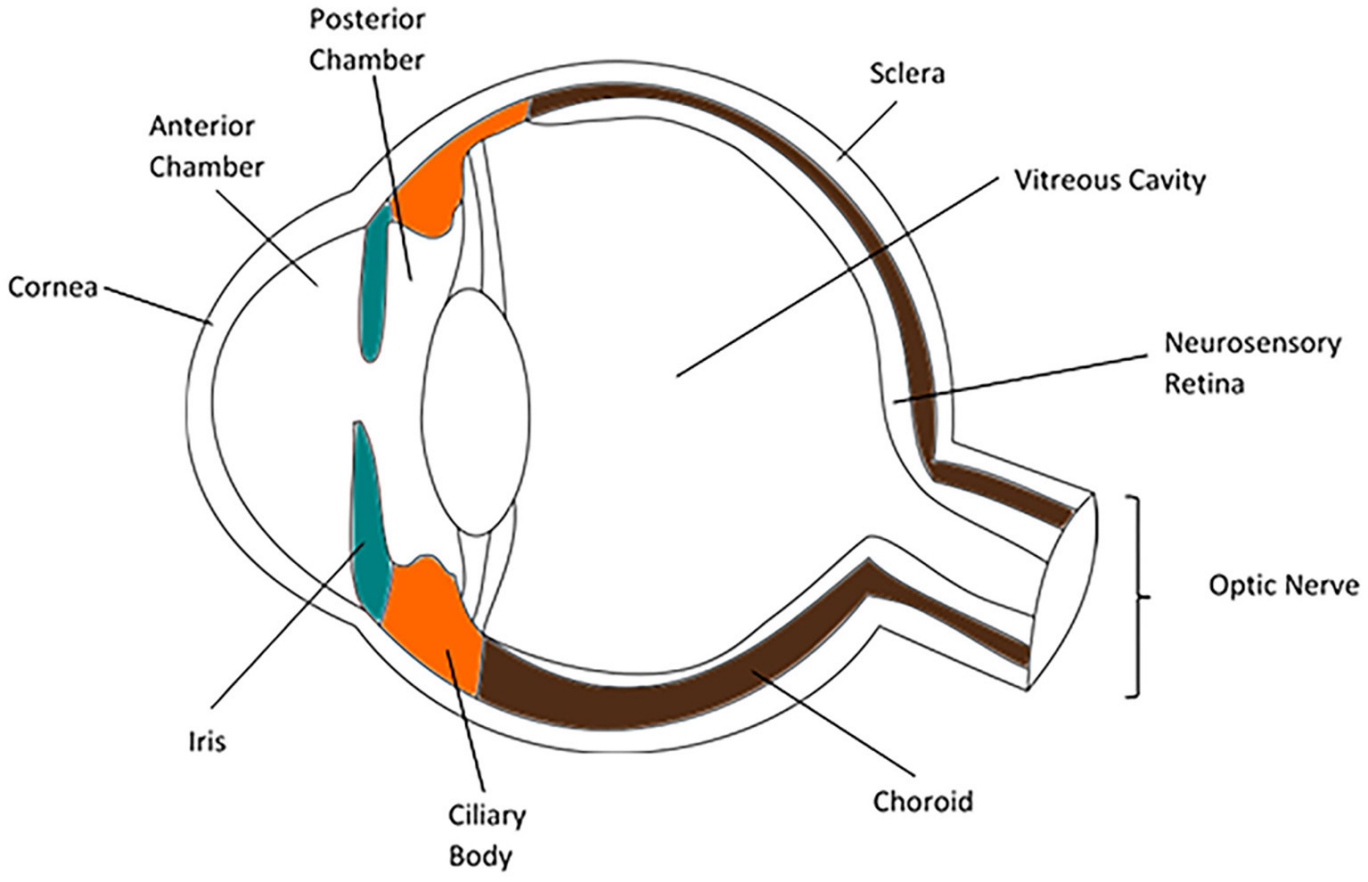
Anatomy of the human eye (3).

The parts of the normal globe (anterior to posterior) are the cornea, anterior chamber, iris (anterior segment), lens, ciliary body, vitreous humor (intermediate structures), retina, choroid, and sclera (posterior segment). Uveitis can be described using this anatomy: anterior uveitis refers to inflammation of focused on anterior chamber; intermediate uveitis involves the vitreous cavity; posterior uveitis affects the retina and choroid; and panuveitis is characterized by the involvement of all layers of the eye (Figure 1) (4).

## Detecting uveitis in the pediatric clinic—The challenge of the red eye

The most common causes of red eye in childhood are ocular surface conditions such viral, bacterial or allergic conjunctivitis, but other causes include ocular inflammatory conditions such as uveitis or inflammation of the sclera, scleritis. It is useful for the pediatrician to understand the “red flags” which warrant timely referral to the ophthalmologist. These comprise photophobia (adverse response to light stimulation) or significant pain, disturbed or reduced vision, and pupil or other structural abnormalities (Table 1). It should be noted that some forms of anterior uveitis are asymptomatic, that unilateral visual loss may not be noted unless vision is checked in each eye separately, and that younger children may struggle to articulate eye symptoms (6, 7). The red eye may be challenging by its absence: “silent” disease in a white (i.e., no redness) eye, which nevertheless has ongoing undetected anterior inflammation, will result in irreversible visual loss, and is unfortunately a common manifestation of JIA associated uveitis.

**Table 1:** Differentiating two key causes of the pediatric red eye* adapted from Gilani et al. (5).

	**Anterior uveitis**	**Conjunctivitis**
Pain	Highly variable, from entirely pain-free, to significant and constant**	Itching/burning sensation
Hyperaemia	Deep	Superficial
Pupil	Normal, constricted or asymmetric	Normal
Aqueous flare (protein exudate within anterior chamber)	Maybe present, and if severe, the turgidity of the anterior chamber fluid may obscure the details of the iris	Absent
Disease course	Relapsing/remitting, typically chronic	Short lived (typically 2 weeks): allergic conjunctivitis may be chronic^¥^

*A newly red eye in a child with a multi-system inflammatory disease known to be associated with uveitis should always be treated with a high index of suspicion.

**Scleritis, in isolation or in association with uveitis, is characterized by severe pain with redness.

^¥^Some auto-inflammatory and autoimmune conditions can cause a chronic primary inflammatory conjunctivitis.

## Making the diagnosis

Uveitis is a descriptive term rather than a diagnosis and may have an infective or non-infective cause. In the following section, we describe the infective causes of uveitis. Note that since there is limited data published on pediatric infective uveitis, some references have been taken from the adult literature.

## Infectious uveitis

Infectious uveitis should be excluded as the cause for childhood uveitis (particularly panuveitis) as the treatment differs significantly from that for non-infectious disease. The details of infections known to cause or be associated with uveitis are mentioned in Table 2. Recommended baseline evaluation for infectious and non-infectious uveitis is highlighted in Table 3.

**Table 2:** Infectious uveitis in children.

**Infection**	**Uveitis description (typical manifestations)**	**Other ocular features**	**Reported from**	**Treatment**	**Prophylaxis required**	**Reference population**
Herpes simplex and zoster virus (8, 9)	- Anterior - Unilateral - Posterior uveitis in neonates with disseminated HSV - Panuveitis in immunosuppressed	- HSV-1 keratitis - Acute retinal necrosis - Unilateral viral conjunctivitis with vesicles on the lid margin	USA	- Oral or topical anti-viral for 7–10 days - Topical steroids Pan or posterior uveitis requires intraocular anti-virals	No	Children
Toxoplasmosis (10–12)	- Congenital or acquired infections - Unilatera - Chorioretinal lesions or scars seen, with new inflammation adjacent to congenital toxo scar - Posterior or panuveitis in children	- Usually, sequalae of congenitally acquired infection	Netherlands Italy USA	Ranges from Pyrimethamine, Sulfadiazine, Clindamycin Azithromycin, prednisone to no treatment as chorioretinitis may be self-limiting in immunocompetent patients	Trimethoprim-Sulfamethoxazole for 45 days in high-risk patients	Children
Syphilis (13–15)	- HIV positive: anterior syphilitic uveitis - HIV negative mainly had panuveitis - Bilateral - Uncommon in childhood but important cause of uveitis with posterior segment changes, as treatment prevents irreversible ocular and systemic sequalae	- Posterior uveitis - Chorioretinitis - Retinal vasculitis	Australia	Moderate duration Penicillin course (between 10 and 21 days)	No	Adults
Tuberculosis (TB) (16–23)	- Granulomatous uveitis - Acute or chronic - Bilateral - Multiple manifestations: Posterior, anterior, intermediate uveitis, panuveitis and retinal vasculitis	- Chronic granulomatous disease is characterized by large “greasy” intraocular inflammatory deposits (mutton fat keratic precipitates), iris nodules, and posterior synechiae	TB endemic countries, e.g., India, China, Philippines, Pakistan, Bangladesh, South Africa, but rising incidence globally	Anti-tubercular treatment combined with systemic corticosteroids	No	Children (through the collaborative ocular tuberculosis study group, COTS)
Cat-scratch disease (24, 25)	Unilateral anterior uveitis most common	- Macular star and optic nerve oedema, neuroretinitis	USA (can be found in any country with cats)	Trimethoprim-Sulfamethoxazole treatment	No	Children
West Nile virus (26, 27)	Multifocal choroiditis	- Optic neuritis, retinitis, neuroretinitis, and occlusive/retinal vasculitis have been reported	USA and India, first reported from Uganda	Supportive therapy and prevention of mosquito bites	No	Adults
Leptospirosis (28)	Acute unilateral or bilateral non-granulomatous panuveitis	- Anterior uveitis, - Panuveitis - Hypopyon - Cataract - Glaucoma Vitreous inflammatory reaction Optic neuritis Retinal vasculitis Retinal hemorrhage	India outbreaks have been reported in Brazil, Nicaragua, Guyana and several other Latin American countries. More in tropical countries.	Doxycycline or penicillin ± steroids	No	Adults and children
Leishmaniasis (29, 30)	2 main phenotypes: - uveitis with active Leishmania infection - Leishmania immune reconstitution syndrome	- Seen more commonly in the immunocompromised	- Mainly South and Central America, Africa, southern Europe and Asia. - 90% severe infections occur in just six countries: Brazil, Ethiopia, Sudan, South Sudan, India, and Bangladesh.	Topical steroids, intra-ocular/sub-conjunctival steroids; systemic steroids; intra-ocular Amphotericin B; systemic amphotericin B; systemic antimonial; systemic miltefosine; pentamidine; systemic fluconazole	No	Adults and children
Ebola virus (31–33)	- Unilateral Anterior uveitis	- Uveitis or conjunctivitis	Sierra leone (West Africa)	Topical or oral steroids	No	Adults and children
Zika virus (32, 34–37)	- Anterior and posterior uveitis reported in infants with congenital infection	- Chorioretinal atrophy, pigmentary mottling of the retina, or optic nerve disease, bilateral macular and perimacular lesions	Brazil and USA	Topical steroids	No	Adults and children
Chikungunya/Dengue (32)	Chikungunya—unilateral or bilateral anterior uveitis is commonest, but all segments of the eye can get involved. Dengue—Posterior and panuveitis initial few weeks and anterior uveitis up to 5 months after onset of symptoms	- Photophobia, retro-orbital pain and conjunctivitis	Endemic in Africa, South and South-East Asia, India, and South and Central America.	Topical, oral or IV steroids based on the involvement of the eye along with cycloplegics	No	Adults and children
Mycoplasma (38, 39)	- Either unilateral or bilateral anterior uveitis	- Intermediate uveitis less common	Israel and Greece	Steroids and anti-mycoplasma antibiotics (erythromycin and amoxicillin)	No	Children
Lyme disease (40–42)	Bilateral chorioretinitis, posterior uveitis and white dot syndrome	- Conjunctivitis, episcleritis, keratitis, uveitis, neuroretinitis, retinal vasculitis and cranial nerve palsies	Turkey, USA and France	Steroids and antibiotics (doxycycline)	No	Adults
Candida (43–45)	Intermediate and posterior uveitis (chorioretinitis)	- Endophthalmitis	Egypt and Netherlands	Oral Voriconazole/Fluconazole, Intravitreal Amphotericin B,	No	Adults
Rubella (46, 47)	Unilateral anterior uveitis	- Keratic precipitates - Iris atrophy and/or heterochromia - Vitreous opacities - Cataract	Netherlands and USA	Topical and systemic steroids, topical NSAIDs and periocular corticosteroid injections	No	Adults
EBV (48–50)	Granulomatous panuveitis	- Chronic active EBV infection associated severe uveitis - Cataract	Japan, China and USA	Valganciclovir and topical or oral steroids	No	Adults and Children
HIV (51, 52)	- Anterior uveitis followed by posterior, panuveitis and intermediate uveitis. - Suspected associations in HIV patients: Herpes zoster, TB or lymphoma or CMV	- Immune reconstitution inflammatory syndrome (IRIS)	UK, Thailand and Czech Republic	Topical steroids, cycloplegics and antivirals	Highly active anti-retroviral therapy (HAART)	Adults
CMV (53–55)	- Chronic and/or recurrent anterior uveitis - Posterior uveitis	- CMV retinitis	Netherlands, Singapore	Valganciclovir or Ganciclovir	No	Adults

**Table 3:** Recommended baseline evaluation of uveitis by the pediatrician.

**Non-infectious uveitis**	**Infectious uveitis**
Inflammatory arthritis associated uveitis -ANA (IIF), HLA-B27 by PCR, ACE (Angiotensin Converting Enzyme)	Bacterial: Mantoux, Interferon Gamma Releasing Assay (IGRA), Chest X ray, Syphilis—VDRL, Mycoplasma IgM
IBD associated uveitis-Fecal Calprotectin	Parasitic: Toxoplasma IgM or PCR, Histoplasmosis, Cat-scratch disease serology, Lyme disease serology
Autoinflammatory causes: genetic testing for recurrent fevers—TINU, CAPS, Blau's disease	Fungal: Candida albicans infection
Vasculitis and SLE associated: p-ANCA, c-ANCA, ANA (IIF), ENA profile, dsDNA, HLA B51	Viral: Rubella IgM, Adenovirus PCR, Herpes PCR, HIV/AIDS, EBV PCR, CMV PCR, VZV PCR
History taking is key: 1. medications/vaccination triggers, 2. review of systems looking for extra-ocular involvement (hearing loss, bowel symptoms, rash, joint pain, morning stiffness, chest pain/shortness of breath etc..)	

### Toxoplasmosis

Globally, Toxoplasmosis is a common parasitic cause of infectious uveitis, and can be either a congenital or acquired infection (the latter being a less common presentation). Eye inflammation can resolve without any sequalae, but often leaves a chorioretinal scaring. In 70–80%, this occurs as a unilateral focal chorioretinal lesion. Reactivation may cause eye pain, blurred vision and can lead to permanent eye damage and blindness. Although serological or intraocular diagnosis is useful, the initial suspicion is based on a typical chorioretinal lesion on eye examination. These lesions can be multiple, solitary or a satellite to an old chorioretinal scar (10–12).

### Herpes viruses

Both herpes simplex and herpes zoster can cause keratouveitis. Unilateral eye disease is more common (Table 2). The presence of cutaneous vesicles, corneal lesions, reduced corneal sensation, and iris atrophy (which may cause unequal pupil shape or size) may be clues to the diagnosis. In acute infections, herpes usually causes non-granulomatous uveitis while in chronic cases granulomatous uveitis is more common (56), with the term granulomatous used to define a clinical phenotype (the presence of iris nodules and large, “mutton fat” inflammatory keratic precipitates) rather than being based on tissue histology. Both herpes simplex and herpes zoster can also cause a retinitis known as acute retinal necrosis. Although rare in children, ARN can cause devastating visual loss. The use of viral PCR on intraocular fluid samples (from anterior or posterior segment) and of long-term systemic antiviral maintenance treatment have improved visual outcomes (8, 9).

### Cat-scratch disease

Bartonella henselae is the principal cause of cat-scratch disease. Although the presentation of ocular inflammation can vary, optic nerve inflammation and macular star (“neuroretinitis”) is characteristic, although the most common initial manifestation is a unilateral anterior uveitis. Exploration of a history of contacts with domestic pets is thus important in childhood uveitis (24, 25).

### Tuberculosis

Ocular TB represents an extrapulmonary form of the disease which may occur in isolation, with no other evidence of pulmonary infection or active disease at any other site in the body (Table 2) (16).

One of the distinguishing features of the TB infection is the development of granulomatous uveitis with the incidence and prevalence depending on the region and endemic nature. Features that should raise suspicion are a history of TB exposure, pyrexia of unknown origin, night sweats, lymphadenopathy, or an immunosuppressed child, and granulomatous forms of ocular inflammation. Bilateral presentation and posterior uveitis are the most reported phenotypes. The Collaborative Ocular Tuberculosis (COTS−1) study reported that chest computed tomography (CT) was three times more sensitive than X ray in identifying old, inactive TB (17). Early anti-TB therapy reduces the rate of recurrences in patients with TB uveitis, whilst systemic corticosteroids reduce the inflammatory sequalae of active disease (18–22).

### Syphilis

Syphilis can manifest in various forms, including posterior uveitis (retinitis, chorioretinitis or retinal vasculitis) and papillitis. The frequency of this uveitis is <1% of all uveitis (13). HIV positive patients had more commonly anterior uveitis as compared to panuveitis or posterior uveitis in the immunocompetent individuals. It is very important to identify syphilis because of its therapeutic implications (14, 15).

### Lyme disease

Eye manifestations occur in 1–4% of infected patients, and whilst early disease can have transient non-specific conjunctivitis, later stages can preset with uveitis, typically intermediate (Table 2) Anterior and posterior uveitis can also occur, as well as keratitis, episcleritis (hyperaemia of the conjunctiva and sclera), exudative retinal detachment and neuro-ophthalmological disorders (40–42). Treatment usually is a combination of antibiotics (usually doxycycline) and steroids (topical, intravitreal, subconjunctival, oral, or intravenous). A history of tick bite may not be present in all patients.

### Candida

Candida infection can cause chorioretinitis and endophthalmitis and may result in visual loss. Endogenous candida endophthalmitis is caused by involvement of the choroid and retina *via* small capillaries, leading to an endophthalmitis with typical “fluff balls” or vitreous abscesses. Other eye findings in endophthalmitis are hypopyon, optic nerve involvement or vitreous abscesses (43, 44). Fluconazale is usually the first line treatment, with new generation azoles such as Voriconazole used previously for resistant cases (45).

### Infectious uveitis in immunosuppressed patients

It is particularly important to exclude infectious causes in the immunocompromised child. The most common infectious uveitides in this population comprise cytomegalovirus retinitis, followed by herpetic acute retinal necrosis, herpetic anterior uveitis, endogenous fungal endophthalmitis, toxoplasmic retinochoroiditis, and herpetic progressive outer retinal necrosis. Intraocular fluid samples are a vital diagnostic tool but visual outcome is usually poor (57).

Human immunodeficiency virus (HIV) may be associated with different types of ocular inflammation caused by various opportunistic or pathogenic viruses, parasites, or bacteria. A retrospective study from Congo reported a prevalence of 14.3% of uveitis in HIV patients. CMV is another frequently associated infection in this group of patients, causing posterior uveitis or retinitis which are considered as one of the most severe ocular complications of HIV associated with severe morbidity (53, 54). The location of ocular inflammation may be predictive of intraocular infection amongst immunocompromised patients as all patients with posterior and panuveitis, had intraocular infections. The most common (almost 50%) may have CMV infection whilst the others may have Toxoplasma gondii, Treponema pallidum, VZV, Aspergillus and Candida. Toxoplasma gondii was most frequently detected amongst non-HIV patients (58).

**Figure 2:**
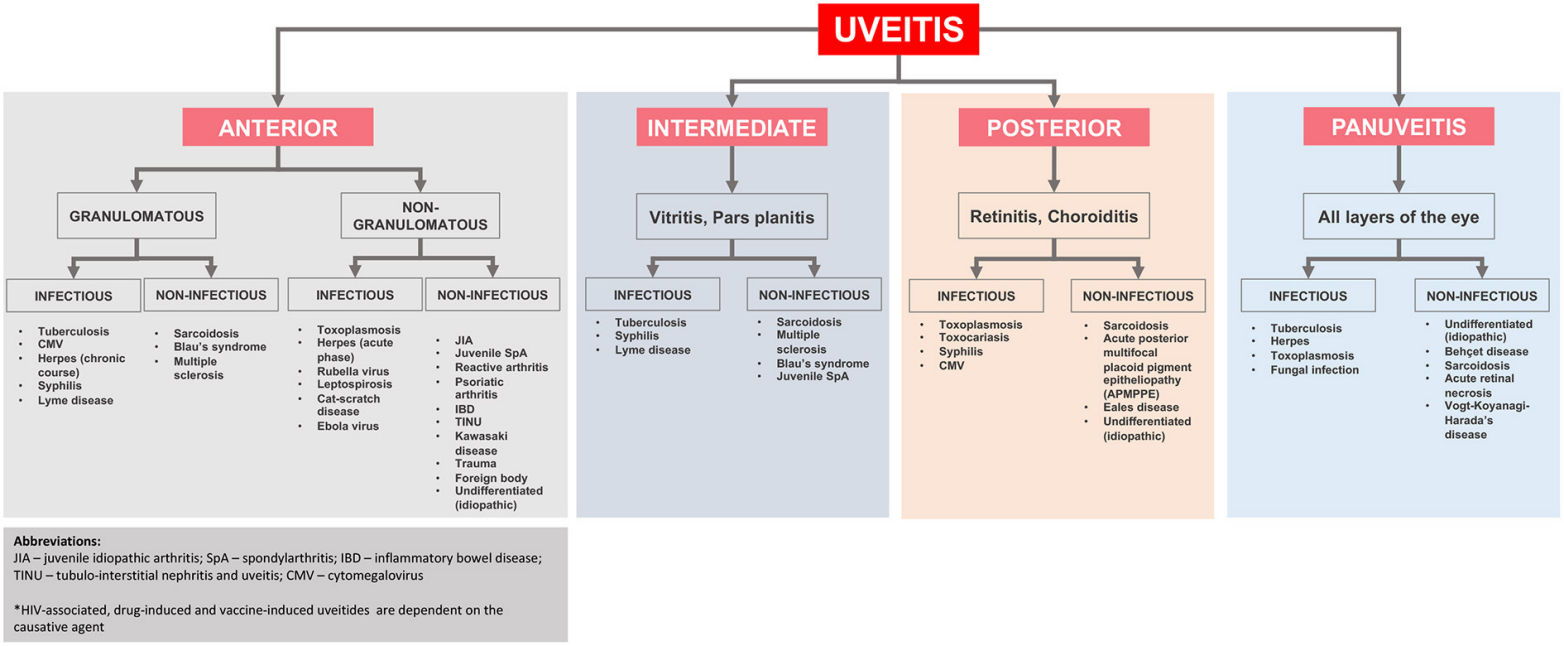
Anatomical classification and underlying diagnoses in pediatric uveitis adapted from Maleki et al. (2).

A simplified flowchart regarding the differential diagnosis of uveitis based on its location and etiology is shown in Figure 2.

## Non-infectious uveitis

### Drug induced uveitis

Drug induced uveitis may be triggered by topical, systemic, or intraocular medications. It is important to take a detailed drug history in patients with unexplained uveitis (59). The onset of uveitis can be immediate or delayed up to several months.

Topical medications such as Brimonidine and Prostaglandin analogs and intraocular injections with Vancomycin, Triamcinolone acetonide or anti-vascular endothelial growth factor (anti VEGF) agents can cause uveitis. Common systemic drugs which may cause uveitis are Cidofovir, Rifabutin, Fluoroquinolones, bisphosphonates, immune checkpoint inhibitors, protein kinase inhibitors and sulfonamides. Mechanism may be toxic or immune mediated (59, 60).

Cidofovir-induced uveitis occurs mostly post-intravitreal injections. Non-granulomatous anterior uveitis and hypotony are two most common findings. Chances of uveitis are higher in patients on protease inhibitors, who have been previously treated for CMV retinitis, have recurrent retinitis, or immune recovery (61). Pathological impact of Rifabutin on the eye is dose dependent and may present as anterior uveitis (with or without hypopyon), panuveitis, intermediate uveitis or retinal vasculitis (62). Treatment with intravenous bisphosphonates (mainly Pamidronate) carries a risk of development of uveitis 1–6 days after administration (63). In such cases, the infusion should be discontinued, changed to a non-nitrogenated bisphosphonate and treatment with topical steroids may be initiated if required (64, 65). Sulfonamides (such as Sulfamethoxazole/Trimethoprim) may cause bilateral anterior uveitis which develops within a week of initiation of therapy (66).

Protein kinase inhibitors have been linked to all types of uveitis and are responsive to topical steroids. Drug-induced Vogt-Koyanagi-Harada syndrome has also been described in association with the use of some protein kinase inhibitors (60).

Antibiotic use and Non-Steroidal Anti-Inflammatory Drugs (NSAIDs) may cause Tubulointerstitial nephritis and uveitis (TINU) which is an immune mediated hypersensitivity reaction. The importance of NSAID as a risk factor varies greatly between the studies but may be present in up to 60–70% of the cases (67).

### Vaccine induced uveitis

There is emerging awareness of a potential link between vaccines and uveitis onset. It is possible that this is not causative, and simply an ascertainment bias with associations only posited because of an incidental temporal association. Post-vaccine uveitis has been described as a mild course of anterior uveitis which are usually responsive to topical steroids. The most common associations are with the Hepatitis B vaccine, but also HPV vaccine, influenza vaccine, BCG, MMR, and varicella vaccine (68–70). Recently, transient uveitis has been described following COVID-19 vaccination with no significant impact on visual outcome (71). A causal link is still debated, and these side-effects of vaccines should not modify the general vaccine calendar.

### Uveitis and systemic diseases (autoimmune and autoinflammatory diseases)

Non-infectious uveitis causes or associated disorders span a wide range of conditions, the most significant of which are discussed here.

#### JIA associated uveitis

JIA-associated uveitis, with a pooled prevalence of 11.8% (95%CI: 11.2–12.4%) amongst JIA patients, is the most common extra-articular manifestation of disease. In comparison, the prevalence uveitis in pediatric SLE is <1% (72). Uveitis prevalence in pediatric Behcets is 18%, but as JIA is the most common rheumatological condition in childhood, JIA is the most common extra-ocular disorder seen in childhood uveitis clinics.

The seven subtypes of Juvenile idiopathic arthritis (JIA) can be divided into JIA with low risk of uveitis (systemic arthritis and other unclassified arthritis), moderate risk (polyarthritis rheumatoid factor, RF, positive) and higher risk of uveitis (oligoarthritis, persistent or extended; polyarthritis RF negative; enthesitis related arthritis or ERA, juvenile psoriatic arthritis or JpSA) (73, 74). Chronic silent anterior uveitis is usually non-granulomatous in oligo or polyarticular JIA, as is acute anterior uveitis of ERA.

Chronic anterior uveitis is the most common form seen in JIA associated uveitis, and in most cases, JIA precedes the onset of uveitis. Children are often asymptomatic at onset and can develop irreversible complications by the time of eventual diagnosis. Complications at diagnosis are highly predictive of later permanent loss of vision, and this underpins the importance of early detection and treatment initiation for JIA-associated uveitis. In the UK and USA, commencement of regular eye examinations is generally recommended to start as soon as possible after JIA is suspected for those at moderate or high risk, and to continue every 4 months until the age of 11–12 years, with the other key time for surveillance being the first few months after stopping systemic JIA treatment.

The risk of developing uveitis appears to be highest in young females with oligoarticular JIA who are ANA antibody positive. As well as differing risk, clinical characteristics differ between JIA subtypes. In sharp contrast to the “silent uveitis” classically seen in early onset oligoarticular JIA, uveitis onset and recurrences in ERA and JPsA JIA can be acutely symptomatic (the “painful red eye”) (75).

Children with juvenile psoriatic arthritis (JPsA, i.e., oligoarthritis, polyarthritis or axial disease with the onset of >4 years of age, and with high prevalence of enthesitis) (76, 77) can also develop an inflammatory conjunctivitis: however, managing pediatricians should have a high index of suspicion for uveitis: 33% of patients with axial involvement were diagnosed with anterior uveitis vs. 6% of the patients with no axial disease (78), and children with JPsA had a worse visual outcome when compared to those with other forms of JIA (79, 80).

Predictors of uveitis incidence in children with JIA include clinical subtype, as above, inflammatory markers, autoantibodies and genetic susceptibility. Several disease activity biomarkers are used routinely (e.g., ESR in JsPA), but there are also emerging biomarkers which are still undergoing validation, including S100A12, S100A8/A9 (also known as MRP8/14) and soluble IL-2 (also known as soluble CD25). The main genetic factor identified in JIA is MHC class II HLA-DRB1 that is involved in antigen presentation being a predictor of JIA or, in some cases, JIA associated uveitis (81).

#### Sarcoidosis

Sarcoidosis can affect almost any portion of the eye or surrounding tissue.

Sarcoidosis associated uveitis can present as anterior, intermediate, posterior or panuveitis. Anterior uveitis may be asymptomatic or painful, but presentation may be with visual symptoms, dependent on the extent, severity and location of ocular inflammation (82). The manifold other examples of ocular disease include dry eye (aqueous deficiency due to infiltration of the lacrimal gland), conjunctival granuloma, scleritis, and optic neuritis.

As well as bilateral and chronic, the uveitis associated with sarcoid is usually clinically granulomatous: large “greasy” keratic precipitates, iris nodules, choroidal granuloma, which may encircle the optic nerve, and/or various degree of vitreous opacities, snowballs and snowbanks in the vitreous cavity (83–85).

Sarcoidosis can be included on the differential diagnosis of almost any presentation of uveitis. Diagnostic criteria for sarcoidosis associated uveitis have been proposed at first International Workshop on Ocular Sarcoidosis (IWOS) in 2009. To make a diagnosis of ocular sarcoidosis, 3 conditions must be met—First, patient must have clinical findings consistent with sarcoidosis by IWOS criteria. Second, evidence of non-caseating granulomas must be demonstrated histologically on a tissue biopsy. Third, other causes of granulomatous inflammation must be excluded such as tuberculosis or syphilis. Many patients with clinical features of ocular sarcoidosis are unable to undergo biopsy or do not have an appropriate site for biopsy. In these cases, a diagnosis of probable or presumed ocular sarcoidosis is made based on clinical features, laboratory findings and chest imaging results according to the IWOS criteria. The IWOS criteria have been recently validated in several countries and shown high reliability for the clinical diagnosis of ocular sarcoidosis (83).

Recommended laboratory investigations in suspected ocular sarcoidosis include chest x-ray to look for bilateral hilar lymphadenopathy, liver function tests, Angiotensin converting enzyme and other sarcoidosis biomarkers in the blood such as lysozyme, neopterin levels and sCD163 serum levels (86).

#### Tubulointerstitial nephritis and uveitis

TINU is a rare benign autoimmune condition of unknown etiology characterized by combined ocular and renal damage. Usually, the eye inflammation precedes or is diagnosed soon after the accidental finding of renal abnormalities and associated systemic features (87–89).

TINU is diagnosed in ~1–2% of pediatric and adolescent patients attending uveitis clinics (90). A Japanese study reported the incidence of TINU at 0.4% among 2556 patients with uveitis (91). Therefore, due to the rarity, most pediatricians, nephrologists and ophthalmologists are unfamiliar with the condition so TINU is often underdiagnosed (89).

Main clinical and laboratory features of TINU include sudden onset of bilateral non-granulomatous anterior uveitis (rare posterior uveitis or panuveitis) associated with typical anterior uveitis symptoms (eye pain, redness, decreased vision, and photophobia) and mildly to moderately abnormal renal function, in particular elevated Creatinine with or without raised urea, high inflammatory markers (ESR, CRP), abnormal urinalysis (low-grade proteinuria, normoglycemic glucosuria, microscopic haematuria and elevated β2-microglobulin). To confirm the renal involvement, kidney biopsy can be considered demonstrating tubulointerstitial inflammatory infiltrations containing lymphocytes and non-specific histiocytes with vessels and glomeruli being typically spared (87, 90, 92). Apart from the mentioned above clinical and laboratory changes, most of the patients with TINU have systemic features, including fever, weight loss, fatigue, and abdominal/flank pain.

Response to treatment is usually good although patients may develop chronic relapsing course of uveitis. Escalation of treatment should be considered without delays to avoid irreversible ocular damage, including, cataract and loss of vision.

#### Vasculitis

Uveitis can be associated with systemic vasculitis. Broadly, pediatric vasculitis is classified as large, medium, small, or variable vessel vasculitis.

##### Behcet's disease

BD comes under an overlapping category of autoimmune and autoinflammatory diseases as both adaptive and innate immune systems play a role in its pathogenesis. The prevalence of BD is exclusively high in countries across the Old “Silk Road”, the area between the Mediterranean and East Asia. The highest prevalence is reported in Turkey, Iran, Israel, Northern Jordan, Korea, Northern China and Japan (93, 94). The most frequent types of ocular involvement in both adults and children are bilateral anterior uveitis with hypopyon, panuveitis, retinitis with retinal infiltrates and occlusive retinal vasculitis with high risk of severe course of ocular inflammation in children (95). The eye is the most frequently involved organ, in up to 60–80% of cases with manifestation within 2–3 years after the onset of BD (94, 96), and in several cases the ocular disease occurs first. This, pediatricians must be aware of the possibility of disease in children with uveitis but must also be aware of the possibility of eye disease in children with BD. Various ocular symptoms may include red eye, floaters, photophobia, and varying degrees of visual loss ranging from mild to severe. The ocular involvement can be recurrent leading to poor visual outcome, so early identification and early and aggressive immunomodulatory treatment approach is essential (97–99).

##### Takayasu arteritis

A type of large vessel vasculitis and defined as panarteritis involving all layers of large vessels of the neck, thorax and abdomen, Takayasu arteritis frequently leads to ischaemic events. The incidence of ocular involvement is rare and includes retinopathy, ischaemic optic neuropathy and much less frequently anterior uveitis. It usually occurs late in the disease course and may be associated with severe involvement of the aortic arch and carotid arteries (100–102).

##### Kawasaki disease

A medium vessel vasculitis, KD is typically associated with bilateral non-exudative bulbar conjunctivitis sparing the limbus and seen in ~85% of children but can also manifest as anterior uveitis in up to 66% of children. Uveitis is usually mild, resolving within 2–8 weeks and maybe associated with keratic precipitates (103, 104).

##### Henoch schonlein purpura

HSP typically presents with a triad of symptoms including palpable purpura, abdominal pain and arthritis. Uveitis is a rare complication of HSP that can be associated with the development of bilateral granulomatous anterior uveitis and keratitis, commonly reported in adult patients or those with HSP-related nephritis (105). The eye disease may be insidious in onset with an aggressive clinical course (106).

##### Granulomatosis with polyangiitis

Orbital involvement is one of the most frequently diagnosed types of ocular manifestations occurring in up to two third of patients with GPA, and in some cases can be the initial presentation of the disease. More broadly, disease tends to affect the respiratory tract and kidneys and is characterized by necrotizing vasculitis of small arteries and veins. A small number of cases of ocular inflammation in GPA can also be associated with uveitis that can be unilateral or bilateral, anterior, intermediate or posterior, with or without vitritis. Granulomatous panuveitis has also been described (107–110).

#### Inflammatory bowel disease

IBD is characterized by chronic remitting-relapsing inflammatory disease of gastro-intestinal tract (GIT) and refers to as Crohn's disease (CD) and ulcerative colitis (UC), where CD is characterized by non-contiguous disturbed, granulomatous inflammation of any part of GIT, while UC is a diffuse, non-specific inflammation of the colonic mucosa, mainly the end part of the GIT, resulting in erosions and ulcer formation (111). The etiological factors are still unknown; however, pathophysiological mechanisms include abnormal response of the host immune system, presence of susceptibility genes and environmental factors (111, 112).

Both, CD and UC have recognized extra-intestinal manifestations (EIMs) that include skin involvement, arthritis (in particular spondylarthritis) and development of ocular inflammation. The muscular-skeletal manifestations may be associated with concomitant eye involvement. The prevalence of eye inflammation is higher in patients with CD than in patients with UC and ranges from 0.6 to 1.8% of all EIMs in the pediatric population (113–115).

Ocular inflammation usually manifests as episcleritis, scleritis but children can also present with a bilateral acute or chronic anterior uveitis. The uveitis can be primary, i.e., manifests before the intestinal symptoms, or secondary (112).

#### Systemic autoinflammatory diseases

SAIDs are a broad spectrum of rare inherited disorders caused by genetic mutations leading to upregulation of key innate immune mediators and characterized by recurrent episodes of self-limiting inflammation without evidence of infection and autoimmunity (i.e., absence of autoantibodies and/or autoreactive effector T cells). SAIDs share common features, particularly recurrent fevers, typical skin rash and multisystemic involvement affecting almost all organ systems depending on the subtype of SAID (116–118).

Depending on the underlying immune mechanisms, SAIDs can be categorized into inflammasomopathies and interferonopathies. Both categories are associated with uveitis, but the most commonly associated SAIDs comprise cryopyrin-associated periodic syndromes (CAPS), familial Mediterranean fever (FMF), TNF receptor associated periodic syndrome (TRAPS), mevalonate kinase deficiency (MVKD), Blau's syndrome (BS), deficiency of adenosine deaminase 2 (DADA2) and haploinsufficiency of A20 (HA20), Aicardi-Goutieres syndrome (AGS), and chronic atypical neutrophilic dermatosis with lipodystrophy and elevated temperature (CANDLE) (116, 119).

According to the literature reports, nearly all SAIDs are associated with transient or chronic recurrent ocular inflammation of varying severity and diversity and in some cases leading to severe ocular impairment. CAPS and Blau's syndrome are more frequently associated with moderate to severe eye inflammation (116).

CAPS is associated with a gain-of-function missense mutation of the NLRP3 gene and include three overlapping clinical phenotypes: familial cold autoinflammatory syndrome (FCAS, mild CAPS phenotype), Muckle-Wells syndrome (MWS, moderate) and neonatal-onset multisystem inflammatory disease (NOMID, severe). Ocular involvement is reported in all phenotypes with conjunctivitis being the most frequently reported, typically occurring during flares. However, a chronic anterior non-granulomatous uveitis is seen roughly in half of CAPS patients, and other uveitis types may develop. Patients with NOMID may also develop papilledema related to high intracranial pressure secondary to CNS involvement (116, 119, 120).

Blau's Syndrome, NFkB activation disorders caused by mutation in NOD2/CARD15 gene, share common clinical features with early-onset sarcoidosis, including recurrent fever, granulomatous dermatitis, sterile arthritis, and ocular inflammation, particularly granulomatours anterior and panuveitis (116, 118, 120).

Ocular inflammation is a recognized feature of the other SAIDS, but occur at a much lower frequency (i.e., found in <1% of the FMF and TRAPS belong to the group of inflammasomopathies that are associated with the mutation in MEFV and TNFRSF1A genes, respectively). The clinical presentation of FMF and TRAPS is similar and characterized by recurrent fever, erysipelas-like or erythematous skin rash, serositis, arthralgia, myalgia, and lymphadenopathy. The most frequent ocular findings are choroidal thickening, retinal vasculitis and anterior uveitis (118, 121–126).

- MVKD is determined by mutation of MVK gene resulting in metabolic defect leading to recurrent fever, rash, painful adenopathy, arthralgia, mouth and genital ulcerations, hepatosplenomegaly, and abdominal pain. MVKD ranges from a milder form known as Hyper IgD syndrome (HIDS), to the most severe phenotype, mevalonic aciduria (MA). The ocular manifestation is less frequently reported with anterior uveitis being the dominant ocular finding, followed by retinal dystrophy and cataract formation in severe cases. The latter is explained by a direct toxicity of mevalonic acid on the retina and lens (116, 118).
- Other rare inflammasomopathies, such as deficiency in ADA2 and interferonopathies, including HA20 haploinsufficiency, Aicardi-Gouttieres syndrome (AGS) and CANDLE can also present with ocular inflammation. This can be associated with severe involvement of choroid, retina, and cornea with high intraocular pressure in some cases (116). Interferon signature is present (upregulation of type-1 interferon regulated genes) and is key in the diagnosis of interferonopathies (127). However, this test is not yet routinely available so clinical features and when possible, molecular diagnosis of SAIDs is most important in suspecting the diagnosis.

#### Reactive arthritis

ReA may also be associated with a various ocular involvement with mild bilateral conjunctivitis being the most frequently diagnosed in the affected patients. The symptoms of conjunctivitis may vary from mild and painless with spontaneous recovery, to severe inflammation including blepharospasm and photophobia requiring topical and sometimes systemic treatment (128). The second most common ocular finding is non-granulomatous anterior uveitis that may occur in up to a third of patients with ReA and more frequently is described in patients carrying HLA B27 and those with sacroiliitis (129).

#### Systemic lupus erythematosus

Ocular manifestation has been described in a third of patients with Juvenile SLE affecting various parts of the eye (130). The most frequently described ocular inflammation include surface epitheliopathy (lacrimal gland involvement leading to keratoconjunctivitis sicca), corneal involvement, episcleritis, scleritis, retinopathy and non-granulomatous anterior uveitis that may lead to blindness. Posterior segment involvement is rare but a reflection of severe systemic inflammation (131, 132).

#### Vogt-Koyanagi-Harada disease

VKHD is described as multisystemic autoimmune sight-threatening disease and is characterized by granulomatous ocular inflammation related to T-cell mediated dysregulation targeting melanocyte-containing tissue in the eye, the CNS, the inner ear, and the skin. The incidence of VKH in pediatric population is rare and race-dependent varying between 0.5 and 3% of all pediatric uveitides (133–136). The challenge in pediatric population is the delayed diagnosis leading to worse visual outcome compared to adults (137).

The etiological factors are still unknown; however viral infection combined with the presence of HLA DRB1^*^0405 may play a role. The high prevalence of VKHD is seen among Hispanic and Asian population with young females being more affected and relatively rare in pediatric cohort (138).

According to the revised criteria proposed by American Uveitis Society (AUS) in 2001, VKHD may present as complete, incomplete, or probable. In its complete form, VKHD is defined as a non-traumatic bilateral panuveitis and is associated with integumentary, neurological/auditory signs. Patients with the incomplete form of the disease usually present with bilateral ocular involvement with either integumentary features or neurologic/auditory manifestations. Probable (ocular) VKHD is characterized by the presence of ocular features with the absence of extraocular manifestations. In all forms of the disease, a history of ocular surgery and clinical or laboratory evidence suggestive of other ocular diseases make the diagnosis of VKH unlikely (139).

Therefore, VKH should be considered if, there is no history of ocular surgery and no clinical or laboratory evidence suggestive of other ocular diseases having similar disease course. There is no single pathognomonic sign or highly specific laboratory tests that could reliably help with the diagnosis of VKHD. Initial step toward the diagnosis of VKHD is a thorough medical history, including history of eye trauma or intraocular surgery, family history of uveitis, detailed systems and medication history.

#### Multiple sclerosis

Multiple Sclerosis (MS) is a chronic inflammatory CNS disorder of the white matter. The reported frequency of MS following uveitis diagnosis ranges from 0.4 to 26.9%. Intermediate uveitis and retinal vasculitis are the most common ocular manifestations, usually occurring bilaterally and most frequently affecting females (140, 141).

#### Pars planitis

Pars planitis is a form of idiopathic intermediate uveitis that primarily affects children. The incidence of pars planitis has been reported to be 1.5–2 cases per 100,000 persons (142) accounting for 5.6–24.0% of pediatric uveitis diagnoses (134). Most cases present bilaterally with floaters and decreased vision. Snowbanks over the pars plana or snowballs in the anterior vitreous are characteristic in the absence of infection or systemic disease (142).

#### Cogan's syndrome

CS is a rare autoimmune vasculitis of unknown etiology that is classically presented with interstitial keratitis and sensorineural hearing loss with tinnitus, vertigo and constitutional symptoms (143). Development of ocular and audiovestibulary manifestations can occur within days or weeks to months. CS can be classified as “typical” characterized by the presence of iritis and conjunctival hemorrhage, and “atypical” in which chronic or recurrent conjunctivitis, scleritis, uveitis, optic disc oedema, and retinal vasculitis are present in addition to or other than interstitial keratitis.

Treatment of CS includes corticosteroids and immunosuppressive agents. It has been proposed recently that early treatment with biologic DMARDs, including TNFα blockers such as Adalimumab and Infliximab or anti-CD20 monoclonal antibody Rituximab, may improve hearing function and significantly reduce eye inflammation (144, 145).

#### Susac's syndrome

SS is a rare self-limiting autoimmune endotheliopathy characterized by the clinical triad: CNS dysfunction, sensorineural hearing loss and retinal vaso-occlusive disease. Branched retinal artery occlusion (BRAO) is the classical ocular involvement in patients with SS resulting in the irreversible visual loss by the occlusions of arterioles supplying the retina. The process can be unilateral or bilateral, characterized by altitudinal defects or central/paracentral scotomas; however, patients may remain asymptomatic if the occlusions are in the peripheral retina (146, 147). Blurred vision and photopsia are most frequently reported features; however, vitreous hemorrhage and neo-vascularisation may also be seen (148).

High dose of corticosteroids and intravenous immunoglobulins (IVIG) are the mainstay of the treatment of SS and have shown high effectiveness. Other treatment options including plasma exchange may be considered as alternative treatment in corticosteroid-resistant cases (149).

### Management

#### Role of the pediatrician in the management and monitoring of pediatric uveitis

Although management of uveitis is led by ophthalmologists, multi-disciplinary care teams which involve pediatricians are needed for holistic care of these inflammatory, chronic, relapsing and remitting disorders (150, 151). They are particularly involved in treatment. The main goals of treatment of chronic non-infectious uveitis are to resolve the intraocular inflammation, to achieve remission and prevent recurrences, to preserve vision and prevent complications (152). Complications include band keratopathy (15.7–29% of all pediatric chronic anterior uveitis), cataract (8–31%), macular edema (6–25%), ocular hypertension/glaucoma (8–19%), ocular hypotension, and macular fibrosis (4%). A systematic review identified that complications of uveitis developed overall in up to 67% of children and one-third of them were present at diagnosis (2, 72).

First line of treatment of anterior chamber involvement is topical steroids, but its use is associated with a risk of ocular complication such as raised intraocular pressure, glaucoma and cataract (153, 154). Long-term treatment is not based on corticosteroids but on disease-modifying antirheumatic drugs (DMARDs) such as Methotrexate as a first line agent. Biologic agents are important for cases refractory to DMARDs.

Pediatric investigations prior to commencement of biologic DMARDs treatment should include work up to rule out tuberculosis (Quantiferon/T-spot, chest x-ray), serology for varicella zoster virus (VZV), hepatitis B, and hepatitis C (and HIV when appropriate). Immunizations should be updated prior to starting immunosuppression, according to national recommendations including live attenuated vaccines such as MMR and varicella vaccine when patient is seronegative (155, 156). Monitoring of compliance with medications and topical treatment is particularly important in children and adolescent patients. Patient with non-infectious uveitis requiring immunosuppression with poor compliance were shown to achieve less steroid free remission (150, 157). Dosing, side effects and monitoring of all these medications are summarized in Table 4.

**Table 4:** Medications and monitoring.

**Medications**		**Dosage used**	**Common side-effects**	**Monitoring**
DMARDs	Methotrexate	−15 mg/m^2^ (up to 30 mg/m^2^/week)	- Gastrointestinal (GI) such as (oral ulcers, nausea, vomiting) - Hepatorenal toxicity	- Liver function - Full blood count
	Mycophenolate mofetil (MMF)	−600 mg/m^2^ twice daily or 2–3 grams/day in divided doses	- Hair loss, fatigue, gastrointestinal discomfort - Leukopenia	- Liver function - Full blood count
	Azathioprine	−2–3 mg/kg/day orally	- Nausea, vomiting diarrhea - Dose related myelosuppression	- Liver function - Full blood count
	Cyclosporine	−2.5–5 mg/kg/day in twice daily dosing	- Hypertension, nausea, vomiting, hirsutism - Nephrotoxicity, hepatotoxicity, anemia, hypercholesterolemia	- Renal and liver function - Blood pressure monitoring
Biologics	Adalimumab	−20 mg Subcutaneous every 15 days for weight <30 Kg - 40 mg subcutaneous every 5 days for weight >30 Kg	- Injection site reaction - Leukopenia	- Pre-biologic screen—(HIV, HepB, HepC, TB) - Full blood count, liver function - Trough drug levels and antibodies
	Infliximab	−5–6 mg/kg IV infusions (up to 10 mg/kg)	- Anaphylactic reaction - Leukopenia	- Pre-biologic screen—(HIV, HepB, HepC, TB) - Full blood count, liver function - Trough drug levels and antibodies

VZV non-immune children who are exposed to chickenpox and not eligible for vaccine should be offered post-exposure prophylaxis (PEP). Although there is no standardized PEP, anti-VZV immunoglobulins (V-ZIG) and oral acyclovir are both used. Varicella immunization of seronegative family members is recommended (158–160). Most immunocompromised patients who develop chickenpox may be admitted for IV acyclovir treatment although data from a single center study showed mostly uncomplicated varicella. However, fever and delay to treatment are predictors of complications (161).

Although there is little evidence in pediatric uveitis, immunizations are safe and effective in children with Rheumatic diseases on immunosuppression but in one third of the patients, response to vaccine may be decreased and this can be confirmed by measuring antibody levels so timing to boosters can be adjusted (162, 163). It is recommended that household contacts of children on immunosuppression are to up to date with immunizations. Children on immunosuppression should follow their normal national immunization schedule for non-live/inactivated vaccines and receive annual influenza vaccine (156). For live attenuated vaccines, in particular varicella and MMR, data suggest these vaccines may still be safe and effective but are usually not recommended in children on biologic DMARDs and systemic corticosteroids (164, 165). Increased disease activity of the underlying disease such as JIA in children on non-biologic DMARDs does not seem to be associated with MMR booster vaccination (164, 166).

Most patients with non-JIA-associated uveitis will have a baseline work-up to identify extra-ocular involvement. The identification of an underlying autoimmune or autoinflammatory disease may tailor management. Extra-ocular involvement may need monitoring that can be undertaken by the pediatrician alongside the tertiary care center. In the absence of extra-ocular involvement at presentation, pediatricians may monitor extra-ocular involvement with history taking, clinical examination, and further investigations as indicated (Table 5). In two recent studies that included children, monitoring identified an underlying disease in 19–29% of the patients monitored for at least 1 year. The most common diagnosis was sarcoidosis and other inflammatory conditions (including JIA, TINU, BD, MS, or Crohn's disease) but also lymphoma and infectious causes (such as tuberculosis, HSV, syphilis or toxoplasmosis). This supports the need for a monitoring based on uveitis classification and evidence (192–194).

**Table 5:** Extra-ocular involvement and monitoring.

**Systemic disease**	**Extra-ocular involvement**	**Monitoring**
**Sarcoidosis** **(****167****–****169****)**	- **General symptoms:** generalized malaise, weight loss, fever - **Upper Respiratory tract**: Rhinitis/sinusitis - **Lower Respiratory tract**: cough, dyspnoea, Interstitial lung disease (ILD) - **Skin manifestations:** Erythema Nodosum, Scar Sarcoidosis, Sarcoid skin lesions - **Lymphadenopathy:** Hilar, Peripheral and Mesenteric lymph nodes - **Organomegaly:** Liver, spleen and kidney enlargement and nephrocalcinosis - **Hypercalcemia and nephrocalcinosis** - **Arthritis:** described typically as “boggy” arthritis - **Glandular involvement:** Parotitis, Salivary gland hypertrophy - **Neurosarcoidosis:** Peripheral neuropathy and CNS involvement - **Uncommon:** Granulomatous bone marrow disease, Hypertension, granulomatous hepatitis, membranous nephropathy, sensorineural hearing loss (SNHL)	- **Chest X ray, Pulmonary function test and HRCT chest:** Lymphadenopathy and ILD, BAL fluid analysis raised CD4:CD8 ratio - **Skin biopsy for diagnosis**: non-necrotising epithelioid cell granulomas - **Ultrasound abdomen and neck:** size and texture of lymph nodes in neck and mesentery, organomegaly, nephrocalcinosis - **Laboratory:** elevated inflammatory markers such as erythrocyte sedimentation rate (ESR) and C Reactive protein (CRP), Serum Angiotensin Converting Enzyme (ACE), serum IL-2R levels, Serum Calcium, Urine Calcium:creatinine ratio - **MRI Brain:** for the diagnosis of Neurosarcoidosis
**Blau's syndrome** **(****170****)** (Typically, a triad of arthritis, uveitis and dermatitis)	- **Arthritis**—Polyarthritis > oligoarthritis (commonly wrists, knees, ankles, PIP), boggy synovitis, tenosynovitis, early onset camptodactyly - **Dermatitis**—polymorphous rash papulo-erythematous, scaly eczematoid, tan colored mildly scaly, ichthyosiform, erythema nodosum - **Non-triad symptoms:** Generalized lymphadenopathy, recurrent fever, ILD, organomegaly, transient facial palsy, large vessel vasculitis, ischemic stroke, nephrocalcinosis, hepatitis, hypertension	- **Based on organ involvement** (Similar to Sarcoidosis) - **Genetic testing for NOD 2 mutation**
**Systemic autoinflammatory diseases** **(****171**, **172****)**	**CAPS: 3 subtypes FCAS**—Cold induced episodes of inflammation, fever, urticaria and joint symptoms **MWS**—Fever, urticarial rash, hearing loss, Amyloidosis **CINCA/NOMID**—more chronic and persistent course, fever, rash, bony overgrowth of the knees, lymphadenopathy, CNS manifestations (severe headaches, mental retardation, chronic meningitis, hydrocephalus and hearing loss) **FMF:** arthritis with erysipelas-like erythema, severe serositis, recurrent fevers **TRAPS:** Myalgia and arthralgia, migratory rash over myalgias, periorbital edema **MVK deficiency:** maculopapular rash, arthralgia, myalgia, severe abdominal pain, diarrhea, splenomegaly, cervical lymphadenopathy, aphthous ulcers, headaches, mental retardation in more severe cases **A20 Haploinsufficiency:** early onset recurrent oral and genital ulcers, abdominal pain and bloody diarrhea, arthritis or arthralgia, episodic fever, and recurrent infections **DADA2** **(****173****):** recurrent stroke with multiple phenotypes but commonly presenting as Polyarteritis Nodosa like phenotype, pancytopenias, organomegaly, leucocytoclastic vasculitis	- **Genetic analysis** for NLRP3 gene - **Monitoring with fever episodes:** inflammatory markers, s. procalcitonin, serum amyloid A, urine routine for proteinuria - **Neurological assessment** for developmental delay - **Hearing assessment for SNHL** - **CAPS DAS** (disease activity scoring) diary to be maintained by the family to monitor each episode - **Fever diary** to be maintained for recurrent fevers
**TINU** **(****174**, **175****)**	**Renal:** Acute tubulointerstitial nephritis, Fanconi syndrome, Nephrogenic diabetes insipidus, Acute kidney injury, chronic kidney disease (kidney symptoms usually precede eye symptoms) **Systemic symptoms**—fatigue, weight loss, fever, anorexia, weakness, and arterial hypertension **Others:** Hearing loss, arthralgia, arthritis, thyroiditis, lymphocytic pulmonary alveolitis	- **Inflammatory markers** ESR, CRP, hypergammaglobulinemia, urinary NAG, urinary Beta 2 microglobulin levels - **Other associations:** hearing assessment, thyroid function, blood pressure monitoring
**Oligoarticular and Polyarticular Juvenile Idiopathic Arthritis** **(****73****)**	**Risk of uveitis:** Extended Oligoarticular JIA (20-30%) as compared to persistent Oligoarticular JIA and RF negative Polyarticular JIA (10-20%). Low risk with RF positive polyarthritis	**Blood tests:** Inflammatory markers ESR, CRP, ANA (IIF), Rheumatoid factor **Outcome measures:** CHAQ for functional assessment of child JADAS−10 or JADAS27 for disease activity JADI for damage assessment
**Juvenile spondyloarthropathy (ERA)** **(****176****)**	**Risk of uveitis:** 5–20% risk of uveitis **Joint involvement:** Axial or peripheral Arthritis or/and enthesitis with inflammatory lumbosacral or sacroiliac pain, dactylitis **Others:** Psoriasis, Inflammatory bowel disease (Crohn's and ulcerative colitis), Atherosclerotic (ischemic heart disease) and non-atherosclerotic cardiac complications (aortitis, aortic valve regurgitation, aortic or mitral valve thickening)	- **Blood tests:** Inflammatory markers ESR, CRP along with HLA B27 - **Outcome measures**: CHAQ, JADAS, BASDAI, ASDAS, and JADI - **Joints:** X ray, Ultrasound or MRI of affected joints - **Associations:** Fecal calprotectin and upper and lower GI endoscopy, Skin biopsy for psoriasis, cardiac assessment by pediatric cardiologist
**Psoriatic JIA** **(****176****)**	**2 distinct subgroups based on joint involvement:** - **Early onset** (1–2 years) like Oligoarticular or Polyarticular JIA predilection for females, ANA positivity, chronic uveitis and HLA DR5 association - **Later onset** (8–12 years) HLA B27 association, more frequent enthesitis, dactylitis, nail pitting and axial involvement. **Skin involvement:** psoriasis, nail pitting, onycholysis and dactylitis	- **Blood tests:** inflammatory markers - **Outcome measures:** functional assessment with CHAQ, JADAS and JADI for disease activity and damage - **Combined multidisciplinary approach** with the Pediatric dermatologist for skin involvement of psoriasis PASI
**Reactive arthritis** **(****128**, **177****)**	- **Seronegative spondyloarthropathy** developing shortly after gastrointestinal, genitourinary or upper respiratory tract infections. - **5 main bacteria associated** are Chlamydia, Salmonella, Shigella, Yersinia and Campylobacter.	- HLA B-27 positivity and sacroiliitis are associated with uveitis - Infectious trigger to be looked for based on history - Chronic or resistant disease to be monitored and treated like ERA
**Inflammatory Bowel disease (IBD)** **(****178****)**	**Dermatological manifestations:** Psoriasis, Erythema nodosum and pyoderma gangrenosum **Rheumatological manifestations:** axial and peripheral spondylarthritis and arthralgia **Bowel involvement:** Crohn's disease or ulcerative colitis	- **Multidisciplinary approach** with the Pediatric dermatologist, Rheumatologist and Gastroenterologist monitoring disease activity and remission. - **Inflammatory markers** ESR, CRP, Fecal calprotectin - **Outcome measures:** CHAQ, JADAS, JADI, ASDAS, BASDAI
**Multiple Sclerosis (MS)** **(****179**, **180****)**	**Acute demyelinating syndrome** causing: - Sensory and motor deficits - Cognitive defects: language, attention, concentration, and memory - More common in adolescent female causing polyfocal deficits	- CSF pleocytosis - EDSS score, cognitive performance, motor performance, neuropsychiatric complaints—fatigue and mood disorders
**Kawasaki disease** **(****181**, **182****)**	**Typical:** Strawberry tongue, cervical lymphadenopathy, polymorphous rash, dry cracked lips, swollen hands and feet, skin peeling, irritability, BCG scar reactivation **Atypical: Cardiovascular**—myocarditis, pericarditis, valvular regurgitation, shock, coronary abnormalities, peripheral gangrene, aortic root enlargement **Respiratory**—pulmonary nodules, peribronchial inflammatory infiltrates **Musculoskeletal**—arthritis and arthralgia **Nervous system**—extreme irritability, aseptic meningitis, facial palsy, sensory neural hearing loss **GI symptoms**—diarrhea, vomiting, abdominal pain, hepatitis, jaundice, gall bladder hydrops, pancreatitis **Genitourinary**—urethritis/meatitis, hydrocele **Others:** desquamating rash in groin, retropharyngeal phlegmon	- Inflammatory markers show raised ESR or CRP with high WBC counts with neutrophilia and high platelets and - 2D ECHO in acute stage for myocarditis and coronary aneurysms (more in subacute stage) - Evaluation based on organ involved
**Behcet's disease (BD)** **(****94****)**	- **Aphthous lesions** in oral and genital mucosa - **Skin lesions:** erythema nodosum, pseudofolliculitis, papulopustular lesions or acneiform nodules - **Musculoskeletal** involvement in 20–40% (usually knees and ankles) self-limited without deformity - **Neurological** involvement called Neuro Behcet's disease—parenchymal or non-parenchymal vascular form - **Gastrointestinal** symptoms, neurological findings, arthralgia, positive family history more common in children as compared to adult BD patients	- **Pathergy test:** Read at 24–48 h - **HLA-B51 test** (50–72% patients with BD are positive) - **Raised inflammatory markers**
**Other vasculitides** **(****183****)**	**Takayasu arteritis:** constitutional symptoms fever, weight loss, systemic hypertension, inflammation of large vessels manifesting with bruits, blood pressure discrepancy in any limb, absent peripheral pulses or claudication, increased inflammatory markers along with angiographic abnormalities of the large vessels **ANCA vasculitis (Granulomatosis with Polyangitis):** upper and lower respiratory tract involvement, histopathological evidence of granulomatous inflammation, laryngotracheobronchial involvement, pulmonary involvement, pulmonary involvement (CT or radiograph), ANCA positivity with or without renal involvement (necrotizing pauci-immune glomerulonephritis) **Henoch Schoenlein purpura:** Palpable purpura or petechiae with lower limb predominance, abdominal pain, intussusception, arthritis or arthralgia, nephritis (haematuria or proteinuria), Histopathology demonstrating IgA deposition	- **Raised inflammatory markers** - **MRA aorta and branches** - **Skin biopsy** of rashes for diagnosis in cases of atypical HSP - **BP monitoring** - **Imaging** of upper and lower respiratory tract - **Monitoring of renal parameters** by serum creatinine, renal biopsy, urine dipstick and urine protein/creatinine ratio
**Vogt-Koyanagi-Harada disease** **(****137****–****139****)**	- **Early-stage disease:** Flu like illness,prodrome of headache, fever, meningismus, confusion - **Late-stage disease:** Tinnitus, vertigo, dysacusis, vitiligo, alopecia and poliosis	- **CSF** Lymphocytic pleocytosis - **Ruling out other differentials** like syphilis, Sarcoidosis and TB
**Juvenile Systemic Lupus Erythematosus (JSLE)** **(****184**, **185****)**	- Chronic multisystemic autoimmune disease symptoms can be divided into constitutional, mucocutaneous, neurological, musculoskeletal, cardiovascular, respiratory, renal, gastrointestinal, ophthalmic and hematological.	- -**Blood tests** ANA (by IIF), dsDNA, Extractable Nuclear Antigen (ENA) Profile, C3, C4, CBC, ESR - **Renal involvement** Urine routine, urine protein:creatinine ratio, BP monitoring - -**Imaging** based on suspected organ involved - **Disease activity scores** SLEDAI 2K, pediatric BILAG scoring for monitoring - **Disease damage scoring** SLICC-SDI
**Cogan Syndrome (CS)** **(****143**, **186**, **187****)**	- Rare autoimmune vasculitis of unknown etiology that is classically presented with interstitial keratitis and sensorineural hearing loss with tinnitus, vertigo and constitutional symptoms - CS is a typical disease of young Caucasian adults with no gender predominance and being very rare in childhood with only a few cases reported in children	- **Exclude differentials** such as infections (syphilis, TB, chlamydia), Sarcoidosis, Polyarteritis nodosa, GPA and Takayasu arteritis. - **Imaging**−2D ECHO and MRI of large vessels - **Monitoring and assessment** of disease by ENT specialist and Ophthalmologist
**Susac Syndrome (SS)** **(****148**, **188****–****191****)**	- Self-limiting autoimmune endotheliopathy characterized by the clinical triad: CNS dysfunction, sensorineural hearing loss and retinal vaso-occlusive disease mainly affecting young adult females, extremely rare in children. - Disease onset may be categorized into two clinical subtypes: either severe encephalopathy associated with headaches, memory loss, psychosis, seizures, dementia, or ocular involvement with recurrent episodes of branch retinal artery occlusion (BRAO) or milder phenotype with even absent neurological features	- **Investigations** include retinal fluorescein angiography (FA), brain magnetic resonance imaging (MRI) and audiometry

CAPS, Cryopyrin associated periodic syndrome; FCAS, Familial cold autoinflammatory syndrome; MWS, Muckle Wells Syndrome; NOMID, Neonatal onset multisystem inflammatory disease; CINCA, Chronic infantile neurologic cutaneous and articular syndrome; DADA2, Deficiency of Adenosine deaminsase 2; TINU, Tubulointerstitial nephritis and uveitis; NAG, N-acetyl glucosaminidase; CHAQ, Childhood health assessment questionnaire; JADAS, Juvenile arthritis disease activity score; JADI, Juvenile arthritis damage index; BASDAI, Bath Ankylosing Spondylitis disease activity index; ASDAS, Ankylosing spondylitis disease activity score; PASI, Psoriasis area and severity index; EDSS, Expanded disability status scale; BILAG, British Isles Lupus Assessment Group; JLSE, juvenile onset systemic lupus erythematous; SLICC-SDI, Systemic Lupus International Collaborating Clinics-standardized damage index; SLEDAI-2K, Systemic Lupus Erythematosus Disease Activity Index 2000.

Close monitoring of children with uveitis is recommended to prevent complications due to uncontrolled inflammation or long-term medication use such as steroids. For JIA-associated uveitis patients, the frequency of screening should be based on individual risk factors (ANA positivity, early age, oligoarticular course) and current immunosuppressive treatment even when the patient is in remission of uveitis (195–197). Similarly for non-JIA associated uveitis, the uveitis screening should continue when remission is achieved although there is no consensus guideline. In most cases monitoring should be done at 2–6-week intervals tailored based on the frequency of topical steroid use, degree of intraocular inflammation, intraocular pressure and presence of complications as agreed by the treating team (195, 198).

### Corticosteroids

Corticosteroids are useful in acute inflammation for non-infectious ocular involvement (199).

Their mechanism of action is by modulating the gene expression of pro-inflammatory cytokines. This anti-inflammatory action of steroids can be achieved either through topical or systemic administration of steroids depending on the location of ocular inflammation. Local treatments include topical steroid drops or periocular, intraocular injections or implants.

#### Topical corticosteroids

Topical corticosteroids are usually the first-line treatment in anterior segment ocular inflammation and were used in about 90% of patients with idiopathic uveitis enrolled in the CARRA Registry. (200). Dexamethasone and prednisolone acetate are the most used topical steroids, the frequency of which is decided by the severity of inflammation. Long-term use of topical corticosteroids (especially difluprednate 0.05%) is associated with cataracts and steroid induced ocular hypertension (201, 202). Literature suggests that the need for >2 drops per day leads to higher ocular complications.

Local injection of corticosteroids is a very useful modality for treating uveitis especially intermediate and posterior uveitis. Regional delivery of steroids is used to minimize systemic side-effects maximizing drug delivery to the target area. This can be done by either periocular (around the eye) or intravitreal (inside the eye) steroid injections or by using intraocular steroid implants.

#### Periocular and intraocular steroid injections

Periocular and intraocular steroid injections are most likely useful for severe presentations or flare up of remitting types of ocular inflammation and for non-chronic uveitis macular edema (203). Ocular side-effects such as glaucoma, ocular hypertension and need for cataract surgery were lesser as compared to intravitreal steroid injections. Intravitreal steroids, used for similar indications as periocular steroids but risks include vitreous hemorrhage, retinal detachment and endophthalmitis.

#### Steroid implants

Steroid implants were created to be used when long-term control of inflammation is desired with minimal relapses. These are approved for treatment of macular edema due to retinal vein occlusion and for treatment of non-infectious posterior uveitis. More studies are required to assess the long-term efficacy and safety profile of steroid implants in patients (204), but they have been used to control posterior disease or as a pre-surgery treatment to lessen the risk of post-cataract surgery macular oedema.

#### Systemic corticosteroids

Systemic Corticosteroids are very useful in children with intermediate, posterior or panuveitis, for urgent control of inflammation. According to the CARRA registry, roughly 94% of children with idiopathic uveitis were given systemic steroids during the disease course (200). Based on severity of inflammation, either oral or IV steroids can be used. Oral steroids are used at a dose of 1–2 mg/kg/day and tapered according to response, while IV steroid boluses are used in more severe cases in higher doses. Recurrences or severe initial presentation may need additional non-biologic or biologic Disease Modifying Anti-Rheumatic Drugs (DMARDs) to limit prolonged steroid associated side effects.

#### Ocular complications of corticosteroids

Ocular complications of corticosteroids are mainly ocular hypertension/glaucoma and cataract. Ocular hypertension and secondary glaucoma have been reported with the use of systemic and topical steroids causing high incidence of blindness at 5 years. Early aggressive immunosuppression with DMARDs to control ocular inflammation may help prevent these ocular complications (205).

Topical Dexamethasone (eye drops) and intravitreal implant (dexamethasone) are both associated with cataract formation and increased intraocular pressure (206, 207). Although there is little data on systemic effects of steroid eye drops in children with uveitis, there are data suggesting that even inhaled steroids affect growth and bone mineral density (208, 209). Systemic corticosteroids have wider side-effects: hypertension, osteoporosis, cataract, gastro-intestinal complications, and metabolic issues (weight gain, diabetes, growth delay). Prevention of weight gain and diabetes may require diet adaptation with limited sugars as well as strict parental control of caloric input. Complications can also have severe long-term adverse events such as peptic ulcers or myocardial infarction (210).

In JIA, growth is known to be affected by disease activity and prolonged high dose systemic corticosteroid use (211, 212). Growth is improved with the use of anti-TNF as it has been shown in several conditions such as JIA or IBD (213, 214). Monitoring of growth is key in patient with non-infectious uveitis as it is likely to be associated with disease activity and the use of steroids. Decreased growth velocity and short stature may play a role in the decision to escalate treatment and even in some selected children to discuss growth hormone therapy, so patient may achieve an acceptable final height.

### Conventional DMARDs

Medicines for long-term immunosuppression are Methotrexate (MTX) (first-line conventional immunotherapy), and less commonly, mycophenolate mofetil (MMF), Azathioprine and cyclosporine A. MTX has a folic-acid analog mechanism of action inhibiting DNA replication and RNA transcription in B and T lymphocytes (199). Folic acid should be used as supplementation to prevent methotrexate related side effects, including anemia, abdominal pain, nausea, vomiting, hair loss. MMF can also be used in refractory uveitis; however, in patients with musculoskeletal involvement, this medication is not known to be effective. Azathioprine and Cyclosporine A are other less commonly used DMARDs used for childhood uveitis and can also be considered in refractory uveitis.

It is well known that withdrawal of Methotrexate may lead to flare of inactive JIA-associated uveitis. A longer period of uveitis inactivity prior to withdrawal may decrease the risk of flare (215).

### Biologic DMARDs

Since pro-inflammatory cytokines, such as TNFα, interferons and Interleukins (IL) are consistently elevated in aqueous fluid and serum of children with intraocular inflammation, targeting these molecules is an attractive and less toxic approach for children with uveitis refractory to steroids and conventional DMARDs. Targeted blocking these pro-inflammatory cytokines can represent a decent steroid-sparing alternative to treat uncontrolled ocular inflammation (216).

#### Anti TNF alpha agents

Adalimumab, Infliximab, Golimumab and Certolizumab are widely used in patients with non-infectious uveitis (217). The effectiveness of Anti-TNF-alpha for treating refractory uveitis has been demonstrated in clinical trials and case series (218–221). Publications show not only a reduction of inflammatory signs and relapses, but also a preserved visual acuity (more than 90% in some cases) along with the quality of life of patients with uveitis (222–224). Early use of anti-TNF is recommended if uveitis is not or is partially responsive to the initial treatment associating systemic corticosteroids and a conventional DMARD to prevent visual loss especially if patient is in a sight-threatening situation. There are also cases when patient is intolerant to DMARDs or is developing ocular and/or systemic side-effects (225, 226). Two main phase III randomized controlled clinical trials (SYCAMORE and ADJUVITE) reporting the use of adalimumab in JIA-associated uveitis have been published in 2017 and 2018. Although using a different design, they both concluded that adalimumab is safe and efficacious in controlling chronic anterior uveitis in patients above 2 years of age who have failed topical steroids and Methotrexate. However, it was shown that the adalimumab group had more side-effect than the placebo group, mainly respiratory infections (220, 221). Etanercept is not efficacious in preventing the occurrence of uveitis and in controlling ocular inflammation in JIA-associated uveitis and is therefore not used in clinical practice for uveitis treatment (217, 227). A small pilot clinical trial did not identify any difference in JIA-associated uveitis disease activity (anterior chamber) in the etanercept group and the placebo group (228).

Although there is limited evidence mostly in JIA associated uveitis, Golimumab and Certolizumab appears to be off-label medications of interest (217, 229–231).

Two systematic reviews identified that adalimumab is better at controlling intra-ocular inflammation in JIA-associated uveitis than infliximab (217, 232). The data from the BiKeR JIA registry showed that there was a tendency for more occurrence of JIA-associated uveitis in the Etanercept group than in the biologic naïve group although the difference was not significant (1.9/100 PY vs. 1.4/100 PY, *p* = 0.09) (233). Similarly, a recent article reporting data from 3 registries, showed that patients with JIA on Methotrexate but also on biologics continues to develop uveitis although at a lower rate suggesting that the use of conventional and biologic DMARDs reduce the incidence of uveitis in JIA (234). Early MTX use and the combination of MTX with an anti-TNF have the highest protective effect (235).

Although limited to few case series, adalimumab and infliximab have been reported to be safe and efficacious in uveitis of several underlying causes including Behcet's disease, Blau's syndrome and sarcoidosis (236–242).

#### Other biologic therapies

Abatacept (CTLA4-Ig), a novel fusion protein that modulates the T cell co-stimulatory signal mediated through the CD28-CD80/86 pathway, Tocilizumab (IL-6 blockade), Rituximab (B cell blockade), Secukinumab (anti IL-17 A monoclonal antibody) are some other off-label agents used in uveitis refractory to the above mentioned therapies (150, 243). Although most of the evidence is in JIA-associated uveitis, it is worth mentioning the use of Tocilizumab with success in non-infectious uveitis with macular oedema (244, 245) and in Behcet's disease as an alternative to anti-TNF agents (246). APTITUDE, a multicentre, single-arm phase II trial, recently assessed the safety and efficacy of tocilizumab in children with JIA-associated uveitis refractory to both MTX and adalimumab. The primary endpoint of the trial was not met but the authors considered that tocilizumab was beneficial in a subset of patients with macular oedema (245).

#### Therapeutic drug monitoring

TDM is not very much used in routine practice for the monitoring of conventional DMARDs such as Methotrexate, Azathioprine or MMF. It is becoming routine practice in the monitoring of biologic DMARDs, particularly anti-TNF. Adalimumab and infliximab are well known to be immunogenic in particular if they are not used in association with a conventional DMARD and therefore monitoring is recommended (247, 248). Therapeutic drug monitoring includes proactive and reactive monitoring. Reactive monitoring is performed when the patient flares so medication dose may be adjusted to achieve clinical remission especially if the level is low and/or if the patient has developed anti-drug antibodies. Proactive monitoring will ensure that the patient's drug level is within the target drug concentration range to allow optimal response. There is a correlation between the drug level and the outcome in the presence of anti-drug antibodies. The presence of anti-drug antibodies may negatively influence the outcome with flares and secondary drug failure. Recent studies were conducted in pediatric inflammatory diseases (247, 249–251). Data suggest that optimisation of anti-TNF levels from initiation of treatment is safe and efficacious in preventing secondary failure in JIA. Using higher dose of a biologic DMARD may prevent the patient from switching to another biologic agent (249, 252). Recent data are also available in JIA-associated uveitis. Anti-adalimumab antibodies are associated with lower drug levels and more active uveitis. The use of concomitant MTX decreased the risk of anti-drug antibodies. In the absence of anti-drug antibodies, a higher drug level was not always associated with disease control (253, 254). A recent clinical trial conducted in adults with inflammatory conditions identified that proactive infliximab monitoring (drug and anti-drug antibody levels) with dose and interval adjustment is significantly associated with sustained disease control with no increased paradoxical adverse events (255). This suggests that TDM is an important tool to achieve optimal disease control.

### Janus kinase inhibitors

There are limited data on the use of JAKi in the management of uveitis. It has been to date only used to rescue patients who failed biologic DMARDs in particular anti-TNF with improvement of the ocular inflammation and with no side-effects (256). A clinical trial is on-going in JIA-associated uveitis and chronic anterior ANA-positive uveitis (257). However, very promising data have been published in other off-labels indications such as Juvenile Dermatomyositis and monogenic IFN-mediated autoinflammatory diseases (258, 259). Tofacitinib has recently been approved in polyarticular JIA following a phase III randomized controlled trial (260).

### Treatment withdrawal

ACR (American College of Rheumatology) and SHARE initiative both recommend at least 2 years of uveitis remission on slit-lamp examination before tapering treatment intensity when uveitis is well controlled on DMARD and biologic therapy only (195, 197). Earlier treatment withdrawal or treatment intensity reduction is at high risk of uveitis flare. The on-going randomized, controlled ADJUST trial's goal is to assess the efficacy of discontinuing adalimumab after achieving JIA-associated uveitis control for at least 12 months (261).

## Impact on quality of life

Visual impairment is an important component of quality of life in children with uveitis and compounds the negative impact of reduced physical function due to associated rheumatological disease (149). Even amongst those with good vision, the fear of blindness is a major stressor for children and their families. Medical interventions including medication side-effects and frequent hospital attendance also contribute to the psychosocial burden of uveitis, as shown by studies involving a patient reported measure (the EYE-Q) developed specifically for children with uveitis. This tool is associated with pedQL and visual acuity (262). EYE-Q scores correlate with extend of uveitis and of visual impairment (263). Early recognition of the psychosocial impact of uveitis and appropriate referral may help families and support patient achieve the best possible outcome (264). It should be remembered that children with uveitis will mature into adults with childhood onset uveitis and it has been shown that on-going active uveitis in adulthood is associated with depression and anxiety (158–160). The number of uveitis related admission in the recent years has significantly decreased likely due to the use of immunosuppressive medications (265). It may be that there is a corresponding reduction in psychosocial burden (266).

## Conclusion

This review on pediatric uveitis presents the case for the multi-disciplinary management of this complex childhood ocular inflammatory disease, in order to improve patient outcomes, patient experience and quality of life. Early recognition of ocular manifestations of established inflammatory disorders reduces the risk of sight loss. In the child who first presents with uveitis, prompt diagnosis of associated systemic conditions is supported by an understanding of the different uveitis types, and in turn supports the individualization of care and early escalation of treatment to reduce morbidity. Management of uveitis, whether it is isolated ocular disease treated with DMARDs, or as part of multisystem disease, involves ongoing surveillance for evolving extra-ocular disease, therapeutic drug monitoring, the management of side-effects of treatment and the psychosocial aspect of loss of vision and the underlying chronic disease.

## Author contributions

All authors listed have made a substantial, direct, and intellectual contribution to the work and approved it for publication.

## Funding

This work was supported by Biomedical Research Center, Great Ormond Street Hospital for Children, London, UK.

## Conflict of interest

The authors declare that the research was conducted in the absence of any commercial or financial relationships that could be construed as a potential conflict of interest.

## Publisher's note

All claims expressed in this article are solely those of the authors and do not necessarily represent those of their affiliated organizations, or those of the publisher, the editors and the reviewers. Any product that may be evaluated in this article, or claim that may be made by its manufacturer, is not guaranteed or endorsed by the publisher.
